# The Influence of Body Mass Index on Outcomes in Patients Undergoing Cardiac Surgery: Does the Obesity Paradox Really Exist?

**DOI:** 10.1371/journal.pone.0118858

**Published:** 2015-03-17

**Authors:** Juan Carlos Lopez-Delgado, Francisco Esteve, Rafael Manez, Herminia Torrado, Maria L. Carrio, David Rodríguez-Castro, Elisabet Farrero, Casimiro Javierre, Konstantina Skaltsa, Josep L. Ventura

**Affiliations:** 1 Hospital Universitari de Bellvitge, Intensive Care Department, IDIBELL (Institut d’Investigació Biomèdica Bellvitge; Biomedical Investigation Institute of Bellvitge), C/Feixa Llarga s/n. 08907, L’Hospitalet de Llobregat, Barcelona, Spain; 2 Department Physiological Sciences II, University of Barcelona, Barcelona, Spain; 3 Department of Public Health, University of Barcelona, Barcelona, Spain; Dasman Diabetes Institute, KUWAIT

## Abstract

**Purpose:**

Obesity influences risk stratification in cardiac surgery in everyday practice. However, some studies have reported better outcomes in patients with a high body mass index (BMI): this is known as the obesity paradox. The aim of this study was to quantify the effect of diverse degrees of high BMI on clinical outcomes after cardiac surgery, and to assess the existence of an obesity paradox in our patients.

**Methods:**

A total of 2,499 consecutive patients requiring all types of cardiac surgery with cardiopulmonary bypass between January 2004 and February 2009 were prospectively studied at our institution. Patients were divided into four groups based on BMI: normal weight (18.5–24.9 kg∙m^−2^; n = 523; 21.4%), overweight (25–29.9kg∙m^−2^; n = 1150; 47%), obese (≥30–≤34.9kg∙m^−2^; n = 624; 25.5%) and morbidly obese (≥35kg∙m^−2^; n = 152; 6.2%). Follow-up was performed in 2,379 patients during the first year.

**Results:**

After adjusting for confounding factors, patients with higher BMI presented worse oxygenation and better nutritional status, reflected by lower PaO_2_/FiO_2_ at 24h and higher albumin levels 48h after admission respectively. Obese patients showed a higher risk for Perioperative Myocardial Infarction (OR: 1.768; 95% CI: 1.035–3.022; *p* = 0.037) and septicaemia (OR: 1.489; 95% CI: 1.282–1.997; *p* = *0*.*005*). In-hospital mortality was 4.8% (n = 118) and 1-year mortality was 10.1% (n = 252). No differences were found regarding in-hospital mortality between BMI groups. The overweight group showed better 1-year survival than normal weight patients (91.2% vs. 87.6%; Log Rank: *p* = 0.029. HR: 1.496; 95% CI: 1.062–2.108; *p* = 0.021).

**Conclusions:**

In our population, obesity increases Perioperative Myocardial Infarction and septicaemia after cardiac surgery, but does not influence in-hospital mortality. Although we found better 1-year survival in overweight patients, our results do not support any protective effect of obesity in patients undergoing cardiac surgery.

## Introduction

Obesity is a risk factor for the development of diabetes mellitus, hypertension and coronary artery disease [1]. Morbid obesity (defined as ≥1.5 ideal weight) and body mass index (BMI) are included in the Parsonnet system and in the Society of Thoracic Surgeons’ model for stratification of the risk for perioperative death [2, 3]. Since mortality risk factors of cardiac surgery and their incidence in obese patients are largely the same as in patients with normal BMI [4, 5], a similar outcome seems likely. However, some reports have shown a better survival rate in overweight and obese patients than in those with normal BMI. This is currently known as the obesity paradox [6, 7]. Indeed, increased BMI alone is not related to an increased perioperative risk in non-cardiac surgery [8] and extreme obesity is not more closely associated with poor survival than normal weight in the intensive care unit (ICU) [9].

On the other hand, obesity is associated with increased morbidity in the ICU. It markedly increases the risk of pulmonary and airway complications [10] and hampers tracheal intubation, mechanical ventilation and weaning, which requires specific ventilatory settings due to the mechanical and inflammatory alterations observed in this condition [11, 12]. In addition, obese patients more frequently develop ICU-acquired infections [13]. As a result, obesity is still regarded as a risk factor for adverse outcomes in the ICU.

The aim of this study was to quantify the effect of diverse degrees of higher BMI on clinical outcomes (morbidity, in-hospital mortality and 1-year mortality) after cardiac surgery and to assess the existence of an obesity paradox in our patients.

## Methods

Data from this prospective study were collected from 2499 consecutive patients undergoing different types of cardiac surgery between January 2004 and February 2009 at our institution. The patients were divided into four groups based on BMI: normal weight (18.5–24.9 kg∙m^−2^; n = 523; 21.4%), overweight (25–29.9kg∙m^−2^; n = 1150; 47%), obese (≥30–≤34.9 kg∙m^−2^; n = 624; 25.5%) and morbidly obese (≥35kg∙m^−2^; n = 152; 6.2%). [1–6]. The underweight group (n = 24) was excluded from the study due to the low number of patients and events. Emergency (n = 124) and redo (n = 265) cases were excluded since they might have introduced a strong bias.

The study was approved by the Institutional Ethics Committee of our hospital (Comité d'Ètica i Assajos Clínics, Hospital Universitari de Bellvitge). Due to the observational nature of the study, the Ethics Committee waived the need for consent. The follow-up was performed using the Catalan Health Central Registry (*Registre Central de Persones Assegurades*, RCA). A complete follow-up for evaluating 1-year mortality was performed in 2379 patients (95.2% of our study population).

Data on and during ICU admission were extracted from the medical registry of each patient in real time using a standardized questionnaire and were entered into a database for analysis. Electronic health records were de-identified prior to use. Recent acute myocardial infarction (AMI) was defined as an AMI that required admission to the hospital during the month before surgery or one that prevented discharge from the hospital before surgery. The other definitions used for this study were based on the Society of Thoracic Surgeons’ national cardiac surgery database definitions [14].

Preoperative data (demographic data, co-morbidities and treatment before surgery), operative data and postoperative variables usually measured on and during admission (including main outcomes) were recorded together with cardiac surgery scores (Parsonnet, European System for Cardiac Operative Risk Evaluation (EuroSCORE) and ICU scores (Acute Physiology and Chronic Health Evaluation (APACHE) II and III, Simplified Acute Physiology Score (SAPS) II and III).

The operations were performed by the same group of cardiac surgeons throughout the study period. Cardiac procedures were performed in all patients using median sternotomy, standard cardiopulmonary bypass (CPB) with moderate hypothermia (34°C) and antegrade cardioplegia. A mean aortic pressure of > 60mmHg was maintained during surgery. Protamine was administered to reverse heparin, in accordance with standard practice. In all patients, decisions regarding postoperative ICU management were made by the attending physician.

### Statistical analysis

Statistical analysis was conducted using PASW statistics 13.0 (SPSS Inc., Chicago, Illinois, USA). Data are expressed as mean ± standard deviation. ANOVA was used to compare differences in characteristics and outcome differences between BMI groups, and a subsequent post-hoc test (Bonferroni test) was used to determine significant differences in the various pairwise comparisons. In all cases, the Kolmogorov-Smirnov test was used to assess the normal distribution of our population and the goodness-of-fit of the final regression models.

A propensity score analysis was used to adjust for preoperative and operative confounding factors in order to minimize baseline differences between the BMI groups. The probability given by the propensity score was included in the different multivariate analyses. A stepwise logistic regression model was used to confirm differences between BMI groups. The BMI groups were included within the models, and the normal weight group was used as control. Survival analysis was carried out with the Kaplan-Meier estimator for the different BMI groups. A proportional hazards Cox regression model was used to evaluate the effect of staging in a BMI group on 1-year survival. The calculation of the statistical power of the results was performed in order to guarantee their appropriateness. A two-tailed *p*-value of 0.05 was considered statistically significant.

## Results

The median BMI of the whole sample was 28.2±4.1 kg·m^−2^ (range 18.7–50 kg·m^−2^). There was a low proportion (0.67%; n = 19) of morbidly obese subjects with a BMI≥40Kg∙m^−2^.

The preoperative characteristics of the patients, including treatment before surgery, are shown in **Table 1**. Univariate analyses showed higher rates of cardiovascular risk factors such as hypertension, diabetes mellitus, dyslipidaemia and hypertrophic cardiomyopathy in higher BMI groups compared with normal weight patients, as well as a higher proportion of treatment on β-blockers, statins and aspirin. Chronic obstructive pulmonary disease rates were higher in the obese groups. Patients in the normal group were likely to be younger than those who were overweight. Haemoglobin before surgery was lower in normal BMI than in the overweight and obese groups. Patients treated with aspirin did not experience a significant increase in postoperative bleeding or requirement for blood products.

**Table 1:** Distribution of preoperative variables according Body Mass Index group.

	Studied group(n = 2449)	Normal weightBMI = 18.5–24.9kg·m^−2^(n = 523; 21.4%)	OverweightBMI = 25–29.9kg·m^−2^(n = 1150; 47%)	Obese BMI = 30–34.9kg·m^−2^ (n = 624; 25.5%)	Morbidly obese BMI ≥ 35 kg·m^2^ (n = 152; 6.2%)	*p*-value
Sex (male/female)	1600 / 849	344 / 179	800 / 350	389 / 235	67 / 85	*0.15*
Age (years)	65.1 ± 11.4	63.8 ± 13.7	65.6 ± 11	65.4 ± 10.2	63.5 ± 10.2	***0.012*** ^A^
Hypertension	64.8% (1588)	46.1% (241)	65.6% (754)	76.3% (476)	77% (117)	***<0.001*** ^A^ ^,^ ^B^ ^,^ ^C^
Diabetes Mellitus	17.9% (438)	13.6% (71)	17.8% (205)	20.2% (126)	23.7% (36)	***0.006*** ^A^ ^,^ ^B^ ^,^ ^C^
Dyslipidemia	52.5% (1285)	39.2% (205)	54.5% (627)	58.5% (365)	57.9% (88)	***<0.001*** ^A^ ^,^ ^B^ ^,^ ^C^
Peripheral vascular disease	9.4% (229)	9.8% (51)	9.4% (108)	9.3% (58)	7.9% (12)	*0.92*
Chronic renal insufficiency	4.7% (114)	5.5% (29)	4.7% (54)	4.2% (26)	3.3% (5)	*0.59*
Renal Failure (on Dialysis)	0.8% (20)	1.3% (7)	0.9% (10)	0.3% (2)	0.7% (1)	*0.69*
Creatinine before surgery (mmol·L^-1^)	95 ± 61	99 ± 81	95 ± 58	93 ± 45	88 ± 56	*0.22*
Previous Stroke	5.2% (127)	5.2% (27)	5.8% (67)	4.2% (26)	4.6% (7)	*0.49*
COPD	12.1% (296)	10.1% (53)	10.7% (123)	15.4% (96)	15.8% (24)	***0.03*** ^B^ ^,^ ^C^
Active smokers	22.9% (562)	26.4% (138)	22% (253)	21.6% (135)	23.7% (36)	*0.45*
Previous Atrial Fibrillation	21.9% (538)	22% (115)	23.5% (271)	19.4% (121)	20.4% (31)	*0.87*
Previous Myocardial Infarction	17% (417)	14.3% (75)	18.4% (212)	16.8% (105)	16.4% (25)	*0.22*
Recent Myocardial Infarction	10.8% (264)	12.2% (64)	10.4% (120)	10.9% (68)	7.9% (12)	*0.45*
On B-Blockers	43.6% (1067)	36.7% (192)	44.3% (510)	46.8% (292)	48% (73)	***0.03*** ^A^ ^,^ ^B^ ^,^ ^C^
On statins	44.6% (1093)	35.4% (185)	45.7% (525)	49.5% (309)	48.7% (74)	***0.001*** ^A^ ^,^ ^B^ ^,^ ^C^
On Aspirin	48.3% (1183)	41.5% (217)	48.5% (558)	52.6% (328)	52.6% (80)	***0.01*** ^A^ ^,^ ^B^ ^,^ ^C^
On diuretics	45.3% (1110)	44.9% (235)	44% (506)	46.2% (288)	53.3% (81)	*0.28*
Hypertrophic cardiomyopathy	33.6% (823)	23.9% (125)	34.9% (402)	37.5% (234)	40.8% (62)	***0.001*** ^A^ ^,^ ^B^ ^,^ ^C^
Dilated cardiomyopathy	20.4% (500)	22.4% (117)	19.8% (228)	21.1% (132)	15.1% (23)	*0.23*
LVEF (%)	60.3 ± 11.8	59.7 ± 13	60.5 ± 11.6	60.7 ± 11.5	60.5 ± 10.7	*0.53*
Hemoglobin before surgery (g·dL^−1^)	13.1 ± 1.6	12.8 ± 1.6	13.2 ± 1.6	13.2 ± 1.7	13 ± 1.6	***0.003*** ^A^ ^,^ ^B^
Platelet count before surgery (1·nL^−1^)	217 ± 69	222± 71	214 ± 66	218 ± 65	224 ± 69	*0.061*
EuroSCORE	5.4 ± 2.5	5.7 ± 2.6	5.4 ± 2.6	5.1 ± 2.4	5.5 ± 2.3	*0.17*
Parsonnet score	10.3 ± 5.9	11 ± 6.4	9.9 ± 5.8	10.3 ± 5.9	10.2 ± 4.8	***0.02*** ^A^

COPD = Chronic Obstructive Pulmonary Disease; NYHA = New York Heart Association classification; LVEF = Left ventricular ejection fraction; EuroSCORE = European system for cardiac operative risk evaluation. Results are expressed as mean ± standard deviation or percentage. Statistical results correspond to ANOVA *p* values. Bonferroni post hoc testing with statistical significant differences:

^A^ between Normal weight and overweight subgroup;

^B^ between Normal weight and obese subgroup;

^C^ between Normal weight and morbidly obese subgroup.

The operative characteristics are shown in **Table 2**. There was a higher proportion of isolated valve surgery in the normal group, and a higher proportion of Coronary Arterial Bypass Graft in the higher BMI groups. Surgeries also included in the analysis were: pericardial surgery 0.8% (n = 18), congenital cardiac surgery 1.8% (n = 45), aortic surgery 2.7% (n = 66), and cardiac tumours 1.4% (n = 35).

**Table 2:** Distribution of intraoperative and postoperative variables according BMI group.

	Studied group (n = 2449)	Normal weight BMI = 18.5–24.9 kg·m^−2^ (n = 523; 21.4%)	Overweight BMI = 25–29.9 kg·m^−2^ (n = 1150; 47%)	Obese BMI = 30–34.9 kg·m^−2^ (n = 624; 25.5%)	Morbidly obese BMI ≥ 35 kg·m^2^ (n = 152; 6.2%)	*p*-value
**Intraoperative data**						
Isolated CABG	35.7% (874)	29.7% (155)	37.4% (430)	38% (237)	34.2% (52)	***0.02*** ^A^ ^,^ ^B^ ^,^ ^C^
Isolated valve	50% (1225)	54.7% (286)	47.5% (546)	49% (306)	57.2% (87)	***0.01*** ^A^ ^,^ ^B^ ^,^ ^C^
CABG+valve	7.6% (186)	8% (42)	8.2% (95)	7.1% (44)	3.3% (5)	*0.35*
Other cardiac surgery	6.7% (164)	7.6% (40)	6.9% (79)	5.9% (37)	5.3% (8)	*0.54*
Number of bypass	2.3 ± 0.8	2.3 ± 0.9	2.3 ± 0.8	2.3 ± 0.9	2.5 ± 0.8	*0.30*
CPB time(min)	110 ± 39	107 ± 37	111 ± 41	112 ± 39	109 ± 33	*0.24*
ACC time(min)	72 ± 29	71 ± 28	72 ± 30	73 ± 28	70 ± 21	*0.59*
**Postoperative data**						
SAPS III	39 ± 10	39 ± 10	39 ± 10	38 ± 10	38 ± 8	*0.29*
APACHE III	48 ± 17	48 ± 17	48 ± 17	48 ± 16	49 ± 15	*0.99*
Ventilation time (hours)	41 ± 113	36 ± 99	35 ± 96	52 ± 135	64 ± 165	*0.07*
Prolonged ventilation (>24 h)	17.8% (438)	15.1% (79)	16.9% (195)	20% (125)	25.6% (39)	***0.001*** ^B^ ^,^ ^C^
PaO2/FiO2 ratio 3 h after admission	327 ± 91	353 ± 93	327 ± 89	310 ± 85	311 ± 107	***<0.001*** ^A^ ^,^ ^B^ ^,^ ^C^
PaO2/FiO2 ratio 24 h after admission	311 ± 73	335 ± 73	312 ± 71	296 ± 71	288 ± 76	***<0.001*** ^A^ ^,^ ^B^ ^,^ ^C^
Reintubation	1% (26)	0.9% (5)	1.3% (15)	0.8% (5)	0.6% (1)	*0.75*
Tracheostomy	1% (24)	0.3% (2)	0.9% (11)	1.1% (7)	2.6% (4)	*0.45*
Need of vasoactive drugs (h)	91 ± 125	88 ± 110	85 ± 107	96 ± 147	113 ± 181	*0.14*
LCOS	39.2% (960)	39% (204)	37.1% (427)	41.7% (260)	45.4% (69)	*0.19*
PMI	12% (293)	11.8% (62)	11.1% (127)	13.5% (84)	13.2% (20)	***0.03*** ^B^ ^,^ ^C^
IABP support	7.5% (185)	6.8% (36)	7.7% (89)	7.7% (48)	7.9% (12)	*0.45*
Atrial Fibrilation	35% (857)	34.9% (183)	36.8% (424)	31.6% (197)	34.8% (53)	*0.37*
Creatinine peak after surgery(mmol·L^−1^)	110 ± 76	112 ± 92	110 ± 75	111 ± 66	102 ± 66	*0.62*
Acute Renal Failure	7.8% (192)	7.1% (37)	7.4% (85)	9.5% (59)	7.2% (11)	*0.38*
Albumin 48h after surgery (g·L^−1^)	28 ± 3.5	27 ± 3.5	28 ± 3.4	28.7 ± 3.7	28.5 ± 3.4	***0.001*** ^A^ ^,^ ^B^ ^,^ ^C^
Hemorrhage-related reexploration	3.4% (83)	3.8% (20)	4.2% (48)	2.1% (13)	1.3% (2)	*0.08*
Pericardial tamponade	0.6% (15)	0.8% (4)	0.5% (6)	0.8% (5)	0	*0.65*
Drainage loss first 12 h (mL)	383 ± 287	392 ± 251	395 ± 307	364 ± 286	344 ± 235	*0.08*
Re-exploration	1.3% (32)	0.6% (3)	1% (12)	2.4% (15)	1.3% (2)	*0.42*
Need for blood products (Units)	1.1 ± 1.7	1.2 ± 1.8	1.1 ± 1.7	1.1 ± 1.5	1 ± 1.7	*0.28*
Stroke	1.5% (36)	2.1% (11)	1.3% (15)	1.3% (8)	1.3% (2)	*0.60*
Septicemia	4% (98)	3.8% (20)	2.6% (30)	5.6% (35)	8.5% (13)	***0.03*** ^B^ ^,^ ^C^
Mean Pre-ICU stay in hospital (days)	6.7 ± 8.5	7.8 ± 9.2	6.8 ± 8.4	6 ± 8.2	6.3 ± 7.6	***0.002*** ^B^
Mean ICU stay (days)	6.6 ± 8.9	6.7 ± 9.3	6 ± 7.1	7.4 ± 11.3	7.8 ± 9.3	***0.007*** ^B^ ^,^ ^C^
Mean hospital stay (days)	23 ± 16.5	24 ± 18	22 ± 15	23 ± 18	24 ± 14	*0.09*
In-hospital mortality	4.8% (118)	5.2% (27)	4.4% (51)	5.4% (34)	3.9% (6)	*0.73*

SAPS = Simplified Acute Physiology Score; APACHE = Acute Physiology and Chronic Health Evaluation; CABG = coronary artery bypass graft; ACC = Aortic cross clamping; CPB = cardiopulmonary bypass. PaO2/FiO2 = Arterial partial pressure of O2 and fraction of inspired oxygen ratio; LCOS = Low Cardiac Output Syndrome; PMI = Perioperative Myocardial Infarction; IABP = intra-aortic balloon pump. Results are expressed as mean ± standard deviation or percentage. Statistical results correspond to ANOVA *p* values. Bonferroni post hoc testing with statistical significant differences:

^A^ between Normal weight and overweight subgroup;

^B^ between Normal weight and obese subgroup;

^C^ between Normal weight and morbidly obese subgroup.

Differences in postoperative data were observed between BMI groups (Table 2). Obese groups (obese and morbidly obese) were more likely to be ventilated >24h, suffer from perioperative AMI and septicaemia compared with the normal weight group. Regarding episodes of septicaemia, the main aetiologies were: deep sternal wound infection 0.04% (n = 1), mediastinitis 0.12% (n = 3), pneumonia 4.8% (n = 12), and bacteraemia 3.3% (n = 82). Higher BMI groups had lower arterial oxygen pressure values and lower fraction of inspired oxygen ratio (PaO_2_/FiO_2_) measurements, without any influence on tracheal extubation or respiratory complications. In contrast, they showed higher albumin blood levels 48h after surgery.

The mean ICU stay was longer in the obese groups than in the normal weight group, while the pre-ICU stay in hospital was shorter in the obese groups. In-hospital mortality was 4.8% (n = 118). Patients died mainly of multiple organ failure (n = 83; 71%), heart failure (n = 29; 23.9%) and septic shock (n = 6; 5.1%). No differences were found regarding in-hospital mortality and its causes. Out of 2379 patients, 2139 survived. 1-year mortality was 10.1% (n = 240).

The differences between groups after adjusting for preoperative and postoperative confounding factors using a propensity score analysis are shown in **Table 3**. No differences in hospital mortality were found between normal weight patients and the higher BMI group. However, the obese group showed higher perioperative AMI and septicaemia compared with normal weight patients. Higher BMI groups were more likely to have worse PaO_2_/FiO_2_ measurements in the first 24h after cardiac surgery and higher albumin blood levels 48h after surgery.

**Table 3:** Final models of multivariable analysis adjusted by a propensity score showing differences between Body Mass Index groups.

	Odds ratio (95%CI)	*p*-value
**Differences between normal and overweight groups**		
Age	1.010 (0.999–1.020)	*0.086*
PaO2/FiO2 ratio 3 h after admission	0.998 (0.997–1.000)	***0.037***
PaO2/FiO2 ratio 24 h after admission	0.996 (0.994–0.998)	***<0.001***
Albumin 48 h after surgery	1.072 (1.031–1.115)	***<0.001***
In-hospital mortality	1.160 (0.564–2.387)	*0.687*
**Differences between normal and obese groups**		
Perioperative Myocardial Infarction	1.768 (1.035–3.022)	***0.037***
PaO2/FiO2 ratio 24 h after admission	0.992 (0.989–0.995)	***<0.001***
Albumin 48 h after surgery	1.132 (1.069–1.198)	***<0.001***
Mean ICU stay	1.020 (0.999–1040)	*0.058*
Septicemia	1.489 (1.282–1.997)	***0.005***
In-hospital mortality	1.146 (0.498–2.635)	*0.75*
**Differences between normal and morbidily obese groups**		
Albumin 48 h after surgery	1.138 (1.051–1.233)	***0.002***
PaO2/FiO2 ratio 24 h after admission	0.991 (0.987–0.994)	***<0.001***
In-hospital mortality	2.435 (0.578–10.249)	*0.225*

**Fig 1:**
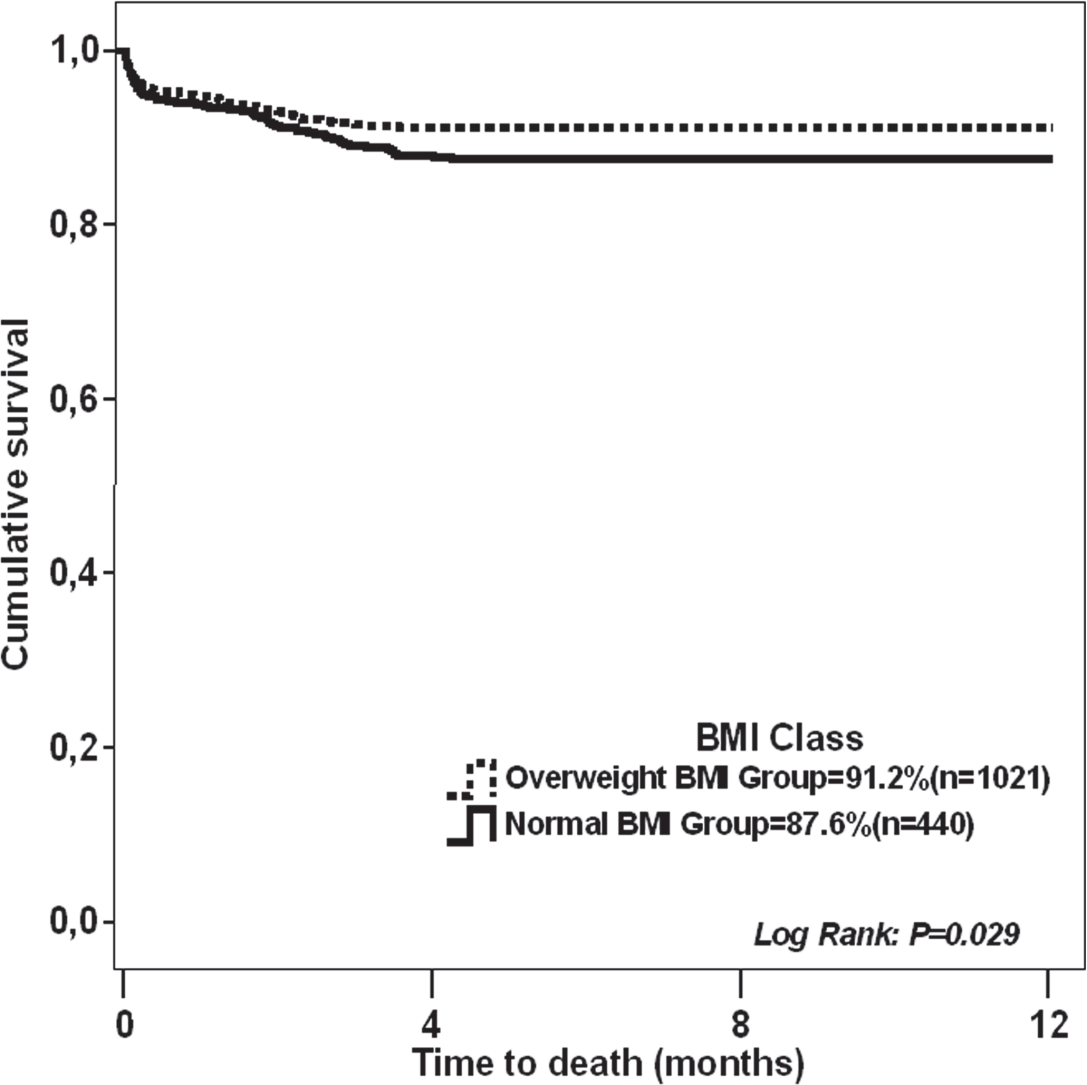
Kaplan-Meier survival curves comparing normal vs. overweight BMI groups.

1-year survival was 89.9%: 87.6% (n = 62/502) for the normal BMI group, 91.2% (n = 98/1119) for the overweight group, 89.7% (n = 63/609) for the obese group and 88.6% (n = 17/149) for the morbidly obese. When we analyzed long-term outcome, the overweight group showed better 1-year survival than the normal weight group when evaluated by Kaplan-Meier survival analysis (**Fig. 1**), and confirmed by a proportional hazards Cox regression model (HR: 1.496; 95% CI: 1.062–2.108; *p* = 0.021).

PaO_2_/FiO_2_ measurements in the first 24h and albumin blood levels 48h after surgery results reached more than 95% statistical power with 95% CI. Results that compare obese group and normal weight patients showed a statistical power of 70–75% and 75–80% for perioperative AMI and septicaemia respectively when we analyzed outcomes in those subgroups.

## Discussion

Previous studies have postulated the existence of an obesity paradox in terms of in-hospital and long-term mortality after cardiac surgery [6, 7, 15], despite the association of obesity with higher rates of cardiovascular risk factors and risk of death in the general population [16]. However, after adjusting for preoperative and operative confounding factors, the present study did not find that obesity had protective effect; nor did obesity exert any influence over in-hospital mortality, despite higher morbidities during the ICU stay in the higher BMI groups, especially in obese patients. On the other hand, overweight patients showed better 1-year survival.

Our findings challenge those of previous studies supporting the obesity paradox after cardiac surgery. In those studies, obese patients tended to be referred for surgical revascularization at a younger age than normal BMI patients due to earlier development of coronary artery disease [6, 7]; this, together with the high levels of care provided in postoperative follow-up, may explain their better survival. Conceivably, the obesity paradox may result from direct comparisons of the study groups which produced a study bias due to incorrect risk adjustments. To minimize the risk of this bias, we performed a propensity score analysis in order to correct for confounding factors [17]. Consistent with epidemiological studies, we found higher cardiovascular risk factor rates in the higher BMI groups, including hypertrophic cardiomyopathy [16]. This condition and obesity itself are associated with diastolic dysfunction and lower coronary microvascular density in the obese population, which may increase the risk of heart failure [18, 19]. In addition, obese patients are more likely to be receiving preoperative statin treatment, which is associated with lower biochemical parameters of inflammatory response and myocardial damage following cardiac surgery [20]. Obese patients tend to receive more aggressive cardioprotective medication regimens, as was the case in our patients, and may also receive improved in-hospital management owing to the perceived increased risk. Thus, all these preoperative factors, together with the particular baseline differences between groups in each population studied and the operative variables shown in the univariate analysis have to be taken into account in order to perform a propensity score analysis. In addition to these conditions, obese patients have a greater number of atherosclerotic lesions in coronary arteries, even at a younger age, than the normal body weight or overweight population [21, 22]. They suffer from low-grade chronic inflammation, which is also related to atherosclerosis and endothelial dysfunction [23, 24]. In consequence, the higher rates of perioperative AMI that we found in the obese might be explained by preoperative risk factors and obesity itself.

Cardiac surgery involves a systemic inflammatory response syndrome with the accumulation of both pro- and anti-inflammatory cytokines, which may lead to a worse outcome [25]. Peripheral adipose tissue has been shown to produce soluble cytokine receptors such as tumour necrosis factor receptors, which are believed to neutralize the harmful effects of cytokines in the myocardium [26]. Lower systemic vascular resistance, plasma renin activity and renin-angiotensin responses, lower levels of atrial natriuretic peptides, and an attenuated sympathetic nervous system are pathophysiological changes in obese patients that can balance the risk that obesity itself represents [27]. This may also explain why overweight and obesity are not associated with higher all-cause and cardiovascular mortality rates in patients with heart failure [28]. At the same time, overweight and obese patients had higher postoperative albumin levels, which may reflect a preserved or increased lean body mass [29]. The correct functioning of the immune response system depends on the metabolic response system, and vice versa. As a result, the overweight and the obese have sufficient nutritional reserve and a more efficient metabolic state, and a better inflammatory and immune response to surgery [30]. This offers a possible explanation for the potential survival benefit in overweight groups in our population.

Higher septicaemia in obese patients is not a new finding. Sepsis is an important risk factor for mortality after cardiac surgery, which produces a sepsis-induced cardiac dysfunction per se [31, 32]. A BMI>30 kg∙m^-2^ has been associated with increased sternal wound infection and saphenous vein harvest site infection [33]. Obesity and diabetes (which itself has a high incidence in obesity) are independent predictors of infection in patients undergoing CABG [34]. Although a high BMI is associated with a higher rate of ICU-acquired urinary tract, pulmonary, ICU-acquired catheter and blood stream infections, it seems to reduce the risk of death from septic shock [13, 35, 36]. Finally, hypoalbuminaemia also increases the risk of infection in cardiac surgery patients [37]. In spite of the lower postoperative albumin levels, normal BMI patients showed lower rates of septicaemia compared with the obese group. We hypothesised that obese patients have higher visceral adiposity, which is associated with chronic inflammation and can aggravate the stress of surgery, leading to an abnormal response in a septic scenario [29, 30].

Obesity is frequently associated with hypoxaemia (reflected by a low PaO_2_/FIO_2_) after CPB [38] and with a longer time on mechanical ventilation in ICU, especially when BMI is ≥40kg·m^−2^ [13]. A lower PaO_2_/FIO_2_ ratio correlated with the time required to carry out extubation and also with lung injury as a marker for outcome in some types of cardiac surgery [39, 40]. In obesity, end-expiratory lung volume is decreased, leading to impairment in the mechanics of the respiratory system, lung, and chest wall, as well as gas-exchange. Thus, recruitment maneuvers added to PEEP are crucial to improve oxygenation and compliance without causing adverse effects on the respiratory function [8, 9]. This may reflect the lack of influence over mortality despite worse oxygenation after admission in the higher BMI groups.

Our study presents certain limitations. The most important is that it was a single-centre observational study. Results related with PMI and Septicemia may seem to be slightly underpowered. However, multivariate analysis still confirm these, even if we take into account that the propensity score equals preoperative factors that may influence in the occurrence of those complications, such as the presence of higher Hypertension rates in the case of PMI and higher *Diabetes mellitus* rates in the case of septicemia. We found a very low incidence of morbidity in obese patients and a relatively low incidence of highly complex surgical procedures which may be due both to the selection bias inherent in a single-centre study and to the optimal postoperative management provided. Among the strengths of this study are the large sample size, which exceeds that of other contemporary studies [7, 15] and the prospective nature of the data. Furthermore, this investigation was conducted at a large tertiary referral hospital with all the patients that underwent surgery with CPB, which makes the study population more homogeneous. In addition, our mortality rates are similar to those reported in other series.

In conclusion, we found higher perioperative AMI and septicaemia after cardiac surgery in our obese population. Worse oxygenation and better nutritional status were also observed in patients with higher BMI, reflected by a lower PaO_2_/FiO_2_ at 24h and higher albumin levels 48h after admission respectively. However, there was no difference between groups in terms of in-hospital mortality. Although we found a better 1-year survival in overweight patients, our results do not support a protective effect of obesity in patients undergoing cardiac surgery. Therefore, our findings do not support the obesity paradox concept, which, in our view, may be the result of study biases. Further studies are needed to examine this issue in greater depth.
